# Subarachnoid Hemorrhage From Ruptured Aneurysms at the Internal Carotid Artery-Posterior Communicating Artery Bifurcation Not Detectable on Preoperative Imaging Studies

**DOI:** 10.7759/cureus.53691

**Published:** 2024-02-06

**Authors:** Akihito Hashiguchi, Kozo Tashima, Takeshi Tonegawa, Koichi Moroki, Hajime Tokuda

**Affiliations:** 1 Neurological Surgery, Tokuda Neurosurgical Hospital, Kanoya, JPN

**Keywords:** undetectable subarachnoid hemorrhage, posterior communicating artery, internal carotid artery, cerebrospinal fluid examination, cerebral aneurysm

## Abstract

Since subarachnoid hemorrhage (SAH) due to the re-rupture of cerebral aneurysms severely worsens the prognosis, an accurate initial diagnosis is essential. Computed tomography (CT) and magnetic resonance imaging (MRI) usually detect aneurysmal subarachnoid hemorrhage (aSAH). However, in rare cases, its identification on CT- and MRI scans is difficult, and a cerebrospinal fluid (CSF) examination is required. We present preoperative imaging and intraoperative findings in patients whose aSAH detection necessitated a CSF examination. Of 225 aSAH patients who underwent preoperative imaging studies at our institution between April 2010 and August 2019, 3 females (1.3%, mean age 57.3 years) harbored undetectable aSAH due to the rupture of an internal carotid artery-posterior communicating artery (ICA-PcomA) aneurysm. The aneurysmal orientation was inferolateral. Intraoperatively, the anterior petroclinoid ligament hampered the detection of the aneurysms that firmly adhered to the surrounding arachnoid membrane. Sustained arterial pulsation and successive minor hemorrhage can lead to the gradual adhesion of an ICA-PcomA aneurysm to the surrounding arachnoid membrane and explain their atypical rupture undetectable on imaging studies and the development of acute subdural hematoma without SAH.

## Introduction

Aneurysmal subarachnoid hemorrhage (aSAH) can be fatal. As aneurysmal re-rupture is likely, patients are at high risk for serious sequelae and death [1,2]. Therefore, aSAH must be diagnosed immediately. SAH is usually easily detected by CT or MRI, but in rare cases, these imaging studies may fail to detect it and cerebrospinal fluid (CSF) examination may be required.

The three cases reported here, all of which were undetected by imaging studies, are SAH due to ruptured internal carotid artery-posterior communicating (ICA-PcomA) aneurysms, and we discuss the anatomical features of these aneurysms and the mechanisms by which they are difficult to detect by imaging studies.

## Case presentation

Methods and case descriptions

Of 225 aSAH patients seen between April 2010 and August 2019, 3 (1.3%) harbored SAH undetectable on imaging studies. All three patients were female, aged 52-66 years (mean 57.3 years), and presented with sudden severe headache or head heaviness, which was not diagnosable on initial imaging studies, necessitating CSF examination. Two patients (case 1, 66 years old; case 2, 54 years old) were seen one hour and three hours after the onset of sudden headache, respectively, both in Hunt and Hess grade I/World Federation of Neurological Societies (WFNS) grade I condition.

Case 1

**Figure 1:**
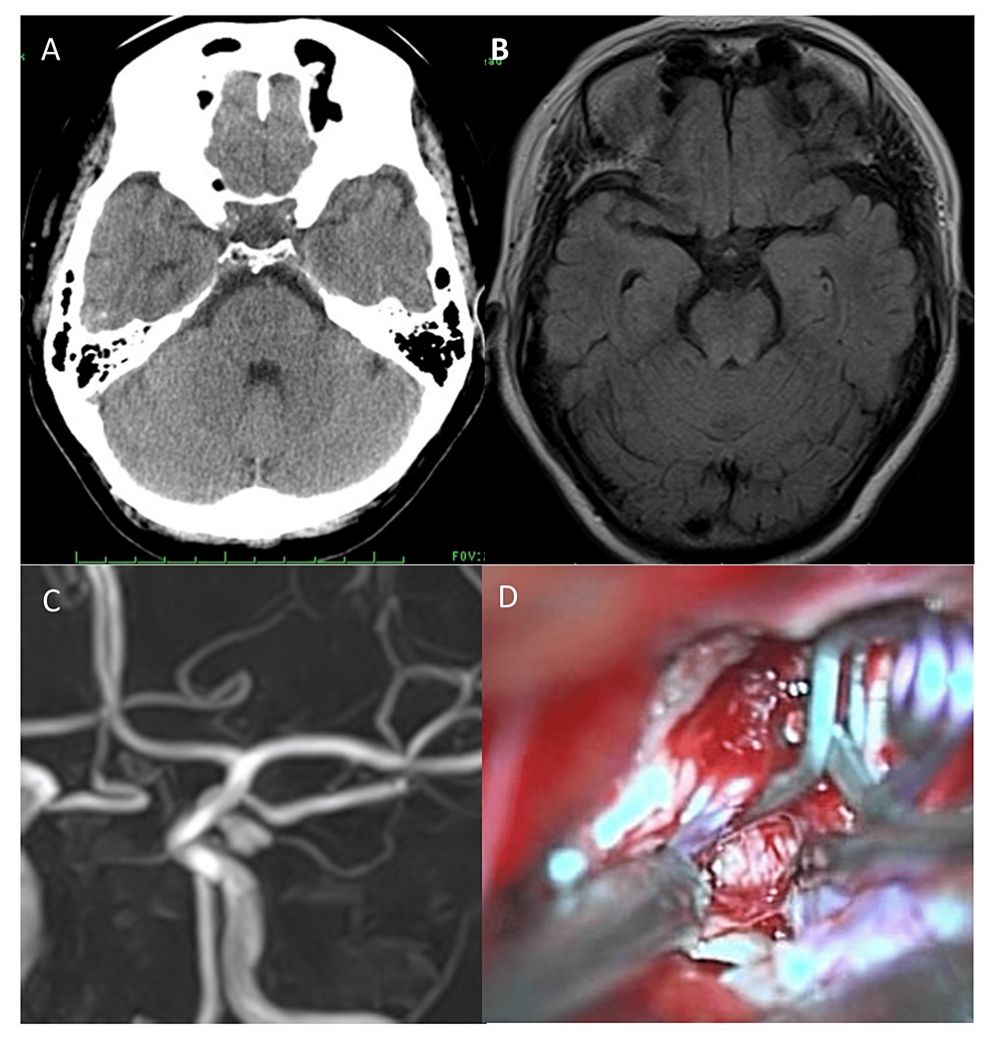
Case 1 Computed tomography (CT, 1A) and fluid-inversion attenuated recovery (FLAIR)-MRI (1B) scans at the initial examination. No SAH was detected. MR angiography (MRA) revealed the inferolateral-directed ICA-PcomA aneurysm with bleb (1C). Intraoperative finding (1D) revealed that the aneurysm firmly adhered at a site behind the anterior clinoid ligament. Partial resection of the anterior petroclinoid ligament was required. SAH: subarachnoid hemorrhage

Computed tomography (CT, Figure 1A) and fluid-attenuated inversion recovery-magnetic resonance imaging (FLAIR-MRI, Figure 1B) returned no findings of SAH (Fisher group 1) [3]. Magnetic resonance angiography (MRA, Figure 1C) identified the ICA-PcomA aneurysm with a bleb at the tip, its diameter was 6 mm, and the direction was inferolateral. The CSF was faintly bloody. Computed tomography angiography (CTA) was performed and the aneurysm was surgically clipped (Figure 1D).

Case 2

**Figure 2:**
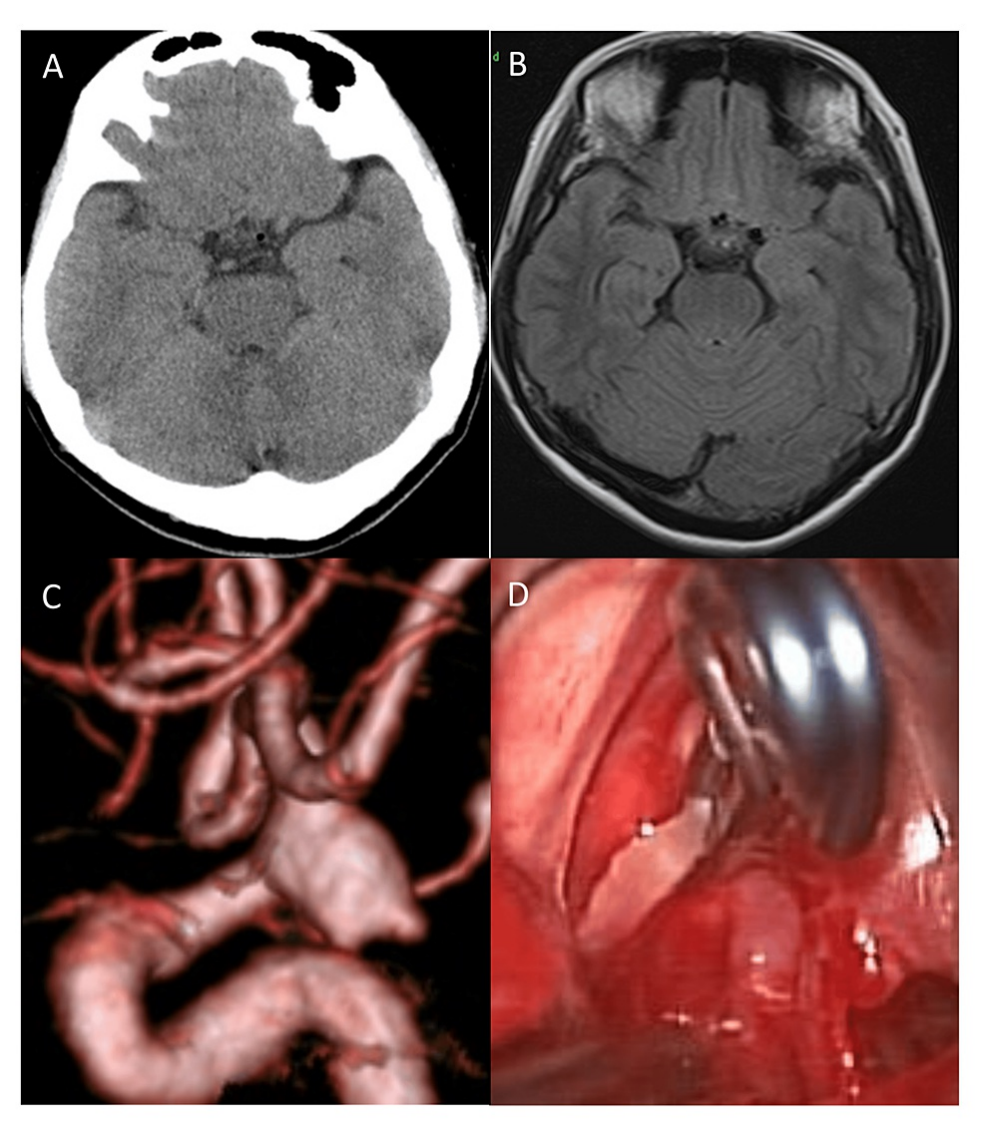
Case 2 CT (2A) and FLAIR-MRI (2B) scans at the initial examination. No SAH was detected. Computed tomographic angiography (CTA) revealed the inferolateral-directed ICA-PcomA aneurysm with bleb (2C). Intraoperative findings (2D) revealed that the aneurysm firmly adhered at a site behind the anterior clinoid ligament. FLAIR: fluid-attenuated inversion recovery; SAH: subarachnoid hemorrhage

CT and FLAIR-MRI returned no findings of SAH (Fisher group 1) [3]. MRA identified the inferolateral-directed, 7-mm-long ICA-PcomA aneurysm with a bleb at the tip. The CSF was faintly bloody. CTA was performed and the aneurysm was surgically clipped. See Figure 2.

Case 3

**Figure 3:**
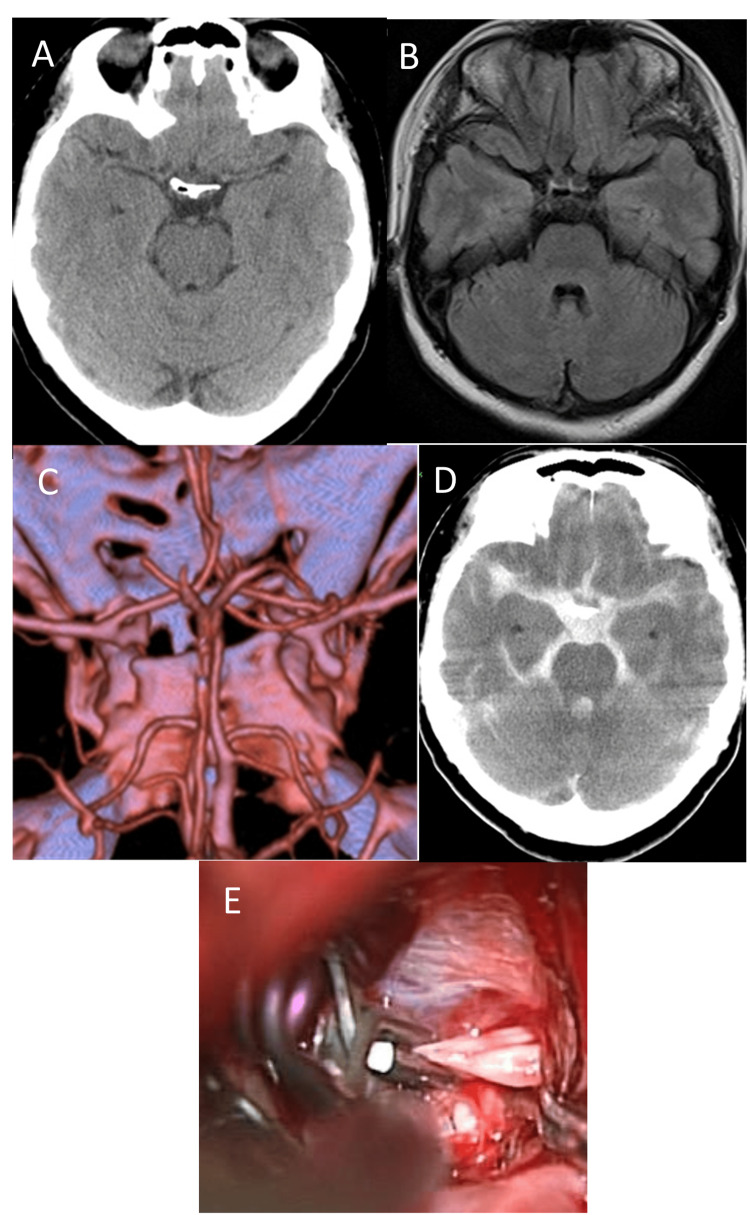
Case 3 CT (3A) and FLAIR-MRI (3B) scans at the initial examination. No SAH was detected. CT angiography (CTA) revealed the inferolateral-directed ICA-PcomA aneurysm with bleb (3C). Shortly after the CTA, the patient became comatose, and a CT scan taken at that time (3D) showed rebleeding (Fisher 3). Intraoperative findings (3E) revealed that the aneurysm firmly adhered at a site behind the anterior clinoid ligament.

This 52-year-old woman had a family history of aSAH; she was admitted on the fourth day after the sudden onset of a sensation of head heaviness (Hunt and Hess grade I/WFNS grade I). While neither head CT- nor FLAIR-MRI scans detected SAH. MRA showed a 6-mm-long ICA-PcomA aneurysm; its orientation was inferolateral. The CSF was faintly bloody. She fell into a coma shortly after the acquisition of CTA images (Hunt and Hess grade 3/WFNS grade 4). Head CT showed Fisher group 3 aSAH [3], and an emergency craniotomy was performed to clip the aneurysm. See Figure 3.

Results

The postoperative course of the three patients was uneventful, their modified Rankin scale (mRS) was 1, and they were discharged home.

Microscopic Intraoperative Findings

On the brain surface of Cases 1 and 2, the CSF was faintly bloody. The trans-Sylvian approach under a surgical microscope detected localized SAH clots around the aneurysms. The orientation of all three ICA-PcomA aneurysms was infero-lateral. The blood flow in the aneurysms was visible through their thin walls. The anterior petroclival ligament hampered the visualization of a portion of the aneurysms. After complete aneurysmal occlusion, we attempted circumferential dissection, however, portions of the wall firmly adhered to and encroached on the surrounding arachnoid membrane. Figures 1D, 2D, and 3D show intraoperative findings in the three patients.

## Discussion

When SAH is not evident on preoperative CT images, gradient-echo T2* or FLAIR-MRI scans may be diagnostically informative [4]. FLAIR-MRI can detect even small SAH clots that are not visible on CT scans. Although the reported sensitivity of FLAIR-MRI is 96.5%, with lumbar puncture as the diagnostic gold standard [5], in our three patients, all of whom presented with the rupture of an ICA-PcomA aneurysm, it failed to detect their SAH.

Since the ruptured aneurysm stops bleeding and the actual amount of extravasated blood in the acute stage of SAH is small, the detection of aSAH is difficult. Upon aneurysmal rupture, a hemostatic aneurysmal thrombus is formed at the rupture site because the arterial blood flow is stopped due to the constriction of the parent artery and compression from extravascular tissues [6-9]. The stiffer the extravascular layer, e.g., the arachnoid lining on the anterior petroclinoid ligament, the stronger the counterpressure exerted by this layer and the easier it is for the bleeding to stop. The three patients we present harbored inferolateral-oriented ICA-PcomA aneurysms whose walls firmly adhered to the arachnoid membrane. Their orientation and strong adherence to the arachnoid membrane hastened the equilibrium between the intra- and extra-aneurysmal pressure and resulted in early temporary hemostasis. Even in patients with a history of aSAH, upon rupture of another cerebral aneurysm, the spread of the new aSAH is limited by the arachnoid adhesion due to the prior aSAH. We suspect this explains the small size and localization of our patients’ aSAH and rendered it undetectable on the initial images.

Although undetectable, SAH and subdural hematomas (SDHs) due to aneurysmal rupture appear to be unrelated, firm adhesion of the aneurysmal wall to the surrounding arachnoid membrane may be implicated. Pure SDH due to aneurysmal rupture is extremely rare. Among 205 cadavers with ruptured aneurysms, only 6 (3%) harbored pure SDHs [10]. Mechanisms proposed to explain their development include successive minor hemorrhage that facilitates aneurysmal adhesion to the arachnoid membrane and results in bleeding from the rupture into the subdural space [11,12]. Intraoperatively, we detected no SDH in Case 3. We think that upon her aneurysmal re-rupture, extravasated blood spread mainly into the subarachnoid space, resulting in a Fisher grade 3 aSAH without SDH. In order, among patients with pure SDH, the rupture site was located at the ICA-PcomA bifurcation (60%), the anterior cerebral artery (ACA) (17%), or the middle cerebral artery (MCA) (13%) [11,13]. Among cerebral aneurysms, ICA-PcomA aneurysms may be the most prone to adhere to the arachnoid membrane because the pulsating pressure elicited in the proximal parental artery is high, and fixation by the dural ring results in vessel immobility. We and others [14] noted that inferolaterally oriented aneurysms tend to be trapped in the anterior petroclinoid ligament. Due to vessel immobility, ICA-PcomA aneurysms tend to adhere to the surrounding arachnoid membrane and even the dura mater.

Although cerebral aneurysms can arise at different sites, only the proximal portion of the ICA and vertebral arteries are anchored to the transdural site. The presence of atherosclerotic changes often observed intraoperatively renders these vessels even less mobile. SDH is more common in patients with ICA-PcomA than vertebral aneurysms because their incidence is higher.

As a limitation, our study population was very small because among 225 patients with SAH due to cerebral aneurysms, only 3 (1.3%) harbored hemorrhages that were undetectable on preoperative imaging studies.

## Conclusions

Regarding the three cases of SAH that could not be detected by imaging studies, we considered that the aneurysms were tightly adherent to the surrounding arachnoid and dura mater, which caused the bleeding to stop early and the volume of the SAH hematoma to be small. Anatomically, ICA-PcoA aneurysms tend to adhere to surrounding structures, which may be why ICA-PcoA aneurysms feature a unique pattern that makes SAH undetectable.
